# Cross-Kingdom Extracellular Vesicles EV-RNA Communication as a Mechanism for Host–Pathogen Interaction

**DOI:** 10.3389/fcimb.2020.593160

**Published:** 2020-11-18

**Authors:** Isadora Filipaki Munhoz da Rocha, Rafaela Ferreira Amatuzzi, Aline Castro Rodrigues Lucena, Helisson Faoro, Lysangela Ronalte Alves

**Affiliations:** ^1^ Gene Expression Regulation Laboratory, Carlos Chagas Institute, Oswaldo Cruz Foundation (FIOCRUZ), Curitiba, Brazil; ^2^ Laboratory for Applied Science and Technology in Health, Carlos Chagas Institute, Oswaldo Cruz Foundation (FIOCRUZ), Curitiba, Brazil

**Keywords:** extracellular vesicles, host–pathogen interaction, bacteria, fungi, infection

## Abstract

The extracellular vesicle (EVs) traffic has been highlighted as a very important pathway of cellular communication. EVs are produced by prokaryotes and eukaryotes organisms and can carry molecules to help maintain homeostasis, responding to general disbalance, infections, and allowing rapid modulation of the immune system. In the context of infection, EVs from both the host and the pathogen have been identified as playing roles in the recruitment of immunological molecules that can lead to the resolution of the infection or the host’s defeat. Bacterial vesicles RNA cargo play roles in the host cell by regulating gene expression and modulating immune response. In fungi the RNA molecules present in EVs are diverse and participate in communication between the host and pathogenic fungi. Little is known about how cross-kingdom sRNA trafficking occurs, although in recent years, there has been an increase in studies that relate EV participation in sRNA delivery. This review aims to elucidate and update the reader concerning the role of extracellular vesicles, with emphasis in the RNA content. We describe the EVs during infection from the host point-of-view, as well as the bacteria and fungi pathogens producing EVs that help the establishment of the disease.

## Introduction

Cell communication is crucial for organisms to maintain homeostasis and respond to adverse conditions, as during an infection. Distinct pathways are involved in cell communication, such as direct cell contact and molecular secretion and the transfer of extracellular vesicles (Raposo and Stoorvogel, 2013). Extracellular vesicles (EVs) are defined as cell-derived particles delimited by a lipid bilayer that cannot replicate and can carry different cargos across the organism; EVs include entities such as apoptotic bodies, exosomes, and microvesicles (Koliha et al., 2016; Théry et al., 2018).

EVs are produced by several organisms, ranging from prokaryotes to mammals and plants (Yáñez-Mó et al., 2015), and every known cell is capable of producing them (Mardahl et al., 2019). The secretion of extracellular vesicles is regulated by various mechanisms that culminate with shedding at the plasma membrane, which can occur spontaneously or in response to certain conditions (Théry et al., 2009). The EV content is closely related to cell-type specificity and is influenced by the physiological or pathological state of the cell (Kalluri and LeBleu, 2020)

Regarding EV classification, there is still no consensus. The difficulty in classifying EVs is attributed to their overlapping sizes, the similar composition of EV subclasses and the lack of knowledge about their biogenesis as well as isolation technique limitations. Currently, EVs are classified into three different subgroups: apoptotic bodies, microvesicles, and exosomes (Théry et al., 2018). Microvesicles and apoptotic bodies are released by plasma membrane budding and their size varies from approximately 50 nm to 1 μm in diameter (Kalluri and LeBleu, 2020). Exosomes have endosomal origin; therefore, they are involved in specific intracellular regulatory processes that determine the exosome content. The size ranges from approximately 40 to 200 nm in diameter (Kalluri and LeBleu, 2020). The biogenesis of exosomes depends on several cell stages (Yáñez-Mó et al., 2015). First, the plasma membrane undergoes invagination associated with soluble molecules from the extracellular milieu, and then, the early-sorting endosome forms. Next, early endosomes mature into late-sorting endosomes, which form multivesicular bodies with intraluminal vesicles in the lumen of the organelle (Yáñez-Mó et al., 2015). When multivesicular bodies fuse with the plasma membrane, they release the exosomes into the extracellular milieu (Hessvik and Llorente, 2018). ESCRT (endosomal sorting complexes required for transport) machinery and tetraspanins are important for exosome biogenesis (Colombo et al., 2013). Their depletion is related to exosome-secretion reduction (Hurwitz et al., 2016; Hurwitz et al., 2017). Other molecules, such as ceramides and sphingomyelinases, are also essential in vesicular transport and are involved in exosome biogenesis processes, such as membrane deformation, fission and fusion (McMahon and Boucrot, 2015). The origin of an extracellular vesicle may be traced by the surface proteins that resemble the cell. Among them is the tetraspanin family, whose proteins are commonly used as EV markers, for example, CD63, CD81, CD9 (Raposo and Stoorvogel, 2013), and the more recently discovered HSP70 (Van Niel et al., 2018). Importantly, so far, there is not a single marker—or a set of markers—that can identify all types of vesicles, and the distribution of tetraspanins among these subtypes is variable (Koliha et al., 2016). The characteristics and composition of EVs differ even depending on the cellular and environmental state (Yáñez-Mó et al., 2015).

The main functions of EVs related to cell communication include molecular transfer, changes in gene expression and cell surface rearrangement (Raeven et al., 2018). In humans, EVs are involved in several physiological processes, such as tissue regeneration (Teng et al., 2015), reproductive biology (Simon et al., 2018) and blood coagulation (Heijnen et al., 2015). They can also act in presenting antigens, stimulating immune responses and tolerogenic effects, immunosuppression, angiogenesis, tumor progression, and the transmission of virulence factors (Raposo and Stoorvogel, 2013). Moreover, they have already been linked with the aging process, cancer (Joncas et al., 2019) and stem cells differentiation (Tatischeff, 2019). Animal and plant pathogen/parasite EV secretion is used to aid survival, cell communication and pathogenesis (Yáñez-Mó et al., 2015). Inside the host, EVs derived from the parasite are related to host modulation, allowing the recruitment of specific immune cells and contributing to the parasite’s life cycle and reproduction (Yáñez-Mó et al., 2015; Dong et al., 2019).

EVs are also studied for drug delivery purposes, due to their biocompatible composition and availability in the organism, as it occurs with encapsulated compounds. The variety of compounds carried inside EVs also makes them attractive in biomarker research, especially in liquid biopsies, which are less invasive and methodologically demanding to execute than other diagnostic procedures (Kalluri and LeBleu, 2020).

Many different biomolecules are carried by EVs, including proteins, lipids and nucleic acids (Yáñez-Mó et al., 2015). In mammalian cells, RNA molecules present in the EVs have been highlighted for their capacity to be internalized by the recipient cell and regulate gene expression (O’Brien et al., 2020). The RNAs identified in EVs include mRNA (messenger RNA), microRNA, rRNA (ribosomal RNA), tRNA (transfer RNA), sRNA (small RNA), and lncRNA (long noncoding RNA) (Im et al., 2018; Turchinovich et al., 2019). Differences in the RNA composition of EVs are based on the cellular state and the producing cell, such as cancer and infection, and can, therefore, be used as biomarkers of certain cellular conditions (Mittelbrunn et al., 2011; Baglio et al., 2015; Shah et al., 2018; Turchinovich et al., 2019). The mechanism that directs the RNA species into the EVs is not understood. The RNA could be passively incorporated due to an RNA abundance in the cytosol; by RNA-binding proteins (RBPs) through motif recognition or RNA secondary structure recognition, and by specific modifications in RNA or RBPs, such as uridylation, ubiquitylation, sumoylation, and phosphorylation (Gibbings et al., 2009; McKenzie et al., 2016; Mateescu et al., 2017; Ragusa et al., 2017).

In this review, we provide an overview of RNAs enclosed in extracellular vesicles, with an emphasis on both the host and the bacteria and fungi pathogens, that are common agents of infection worldwide.

## EVs and Infection: The Host Response

In an infectious environment, host-originated EVs can promote both immunostimulant and immunosuppressive events; they can carry antigenic molecules as well as Major Histocompatibility Complex (MHC) molecules (Mardahl et al., 2019). Antigen presenting cells (APCs) are at the center of studies involving host-originated extracellular vesicles. They are believed to mediate interactions with T and B cells to: a) activate naïve T cells through MHC molecules, b) serve as exogenous antigens for APCs, c) present antigens directly to CD4+ T cells, and d) present antigens to dendritic cells (DCs), which will be loaded into their own MHCs. EVs originating from host cells have also been reported to inhibit IL-8 and TNF, reducing inflammation. Additionally, the immune response can be promoted by EV content through antigen-independent mechanisms (Mardahl et al., 2019; Raposo and Stahl, 2019). On the other hand, EVs can mediate protective messages between cells during stress situations (Tatischeff, 2019). In a model of *Conidium* infection, the authors identified different EV cargo compositions derived from PMN cells enriched with antimicrobial molecules to respond to the infection (Shopova et al., 2020). It has been shown that the microRNA signature of EVs is different from the in-microRNA signature of the cell (Mittelbrunn et al., 2011; Roderburg et al., 2013). In the context of the immune system, some micro-RNAs, like miR-760, miR-632, miR-654-5p, and miR-671-5p, were loaded into activated T cell EVs and they were more abundant in the EVs compared to the parental cell during immune synapsis (Mittelbrunn et al., 2011). Additionally, vesicles from different species can lead to distinct protection against diseases. For example, EVs from *Nippostrongylus brasiliensis*, a hookworm that causes chronic gastrointestinal infection in humans, were isolated and injected in mice. After the EV injection, colitis was induced and the EVs protected the animals against the inflammation. However, EVs from the whipworm *Trichuris muris* did not present the same protective pattern. From RNA-Seq data of *N. brasiliensis*, 52 differentially expressed microRNAs were identified, and it was shown that they play an important role in inflammatory cytokine suppression and secretion of anti-inflammatory IL-10 in the host cell (Eichenberger et al., 2018).

Within the gut microbiome, host vesicles have been reported to enter microorganisms and regulate their gene expression and growth (Lee, 2019). The gut microbiota is composed of 100-200 distinct bacterial species. Depending on genetics, diet and disease state this composition may vary, and a series of correlations have been described regarding the microRNA-mediated control of the gut microbiota. Liu et al. (2016) identified the most abundant miRNAs present in the feces of human and mice and compared to those observed inside the Evs. The most abundant miRNAs observed in the EVs were miR-1224, miR-2146, miR-2134, miR-483, miR-710, miR-2141, miR-720, miR-155, and miR-34c. The authors showed that some miRNAs were able to enter the bacterial cells and regulate gene expression by RNA alignment. The host miRNA targeted rRNA and ribozyme (RNaseP), but the expression could be induced or repressed, depending on the species analyzed. In addition, using mice defective in the miRNA pathway (Dicer knockouts), it was shown that the KO mice were more susceptible to induced colitis than the Wild type and that fecal miRNA transplantation could help to restore the gut microbiota (Liu et al., 2016). The EV traffic also helps communication between components of the microbiota through biofilm formation or aggregation-derived quorum sensing, promoting the homeostasis of the host and even prevent and fight infections (Morales and Hogan, 2010). The quorum sensing process uses extracellular signals to communicate and coordinate social activities. In *Pseudomonas aeruginosa* the molecule PQS (Pseudomonas quinolone signal) is an important quorum sensing molecule that is transported into vesicles. When this molecule was removed, it led to problems with cell-cell communication (Mashburn and Whiteley, 2005).

EVs are recognized as important players in the pathological process of sepsis, influencing aspects such as coagulation and hyperinflammation disturbances. A study showed that several microRNAs were dysregulated in septic shock, highlighting exosomal miR-125b-5p that was validated as a survival predictor and miR-26b-5p and miR-199b-5p, which were able to differentiate healthy individuals from septic patients (Reithmair et al., 2017). Bacterial vesicles were introduced intraperitonially in mice that afterwards presented symptoms of sepsis-like inflammation and eventually died, highlighting that the EVs were sufficient to trigger the host inflammatory response (Park et al., 2018). Additionally, it has been described that the pathogen vesicle formation can be affected by the antibiotic choice in the treatment of sepsis—a great contributor to treatment success—making it even more important to understand the phenomena surrounding the EV-cargo-microbe-host system (Dagnelie et al., 2019).

In addition to the host cell response to infections, one must consider the role of the EVs being shed by the pathogens; the next topics are focused on the EVs produced by common pathogens identified in worldwide infections—bacteria and fungi.

## Microorganisms—EV Shedding and Their Roles in Cell Communication and Disease

### Bacterial Membrane Vesicles

The bacterial membrane vesicles (MVs) are composed of a lipid bilayer and have a size ranging from 20 to 400 nm. As described for eukaryotes, there are different MV categories, varying according to their structure, composition and origin (Toyofuku et al., 2019). The outer membrane vesicles (OMVs) are a class of vesicles produced by gram-negative bacteria, derived from the outer membrane, and due to its origin, they are covered by lipopolysaccharides (LPS) (Schwechheimer and Kuehn, 2015). The OMVs biogenesis is still unclear, however some models are proposed to explain the process. One of these models is based on the dissociation of the covalent linkage between the outer membrane and the peptidoglycan layer, being the absence of these bonds associated with the growth of the outer membrane leading to the formation of OMVs (Kulp and Kuehn, 2010; Schwechheimer and Kuehn, 2015). Another model proposes that OMVs are formed by protuberances that appear on the outer membrane due to an increased pressure in the periplasmic space caused by the accumulation of misfolded proteins and fragments of peptidoglycans (Kulp and Kuehn, 2010; Schwechheimer and Kuehn, 2015). A third model is related to an enrichment of curvature-inducing molecules, such as quinolone PQS of *P. aeruginosa* (Mashburn-Warren et al., 2008). Roier and coworkers proposed a novel method based on phospholipid accumulation as a result of deletion or downregulation of *vacJ* and/or *ybr* genes (Roier et al., 2016). More recently a group of genes involved in OMVs biogenesis in *Salmonella enterica* Serovar Typhi were identified (Nevermann et al., 2019). This group contains some genes related to envelope stability, LPS synthesis, peptidoglycan synthesis and remodeling, stress sensor and transcription regulator. Gram-negative bacteria also produce outer-inner membrane vesicles (OIMVs), that are originated from the inner membrane and were firstly observed in *Shewanella vesiculosa* M7T and other pathogenic bacteria (Pérez-Cruz et al., 2013; Pérez-Cruz et al., 2015). These OIMVs contain both the outer membrane and the inner membrane, as well as cytoplasmic components. Gram-positive bacteria also produce MVs; however, the mechanisms of generation and release through the cell wall are not well known (Brown et al., 2015; Liu et al., 2018). Although the internal content is similar, the MVs of gram-positive and gram-negative bacteria have different glycoconjugates (Gill et al., 2019).

Several functions are attributed to bacterial MVs, including communication with other bacteria. The distribution of antimicrobial resistance genes is an example of this interaction, being considered a type of horizontal gene transfer (Chatterjee et al., 2017; Domingues and Nielsen, 2017). The presence of DNA in MVs has been reported in several cases, and even the acquisition of resistance in sensitive bacteria has been observed after exposure to OMVs from resistant bacteria in *E. coli* (Kim et al., 2018). MVs can also mediate the host cell invasion process and act in competition with other pathogens, due to the presence of several virulence factors and toxins. In *P. aeruginosa* OMVs, multiple virulence factors that are involved with the host colonization process were identified, such as β-lactamase associated with host peptide degradation, alkaline phosphatase involved in biofilm formation, hemolytic phospholipase C, and Cif related to *P. aeruginosa* virulence (Bomberger et al., 2009). Toxins involved in cell death induction were found in OMVs from enterohemorrhagic *Escherichia coli* (EHEC) O157, such as cytolethal distending toxin V and EHEC-hemolysin (Bielaszewska et al., 2017). In addition, MVs can induce the host immune response through interaction with pathogen-associated molecular pattern (PAMP) receptors. Molecules such as LPS, peptidoglycan, lipoprotein, DNA, and RNA are recognized by host cell receptors, such as Toll-like receptors (TLRs), resulting in the induction of signaling cascades and the production of proinflammatory molecules (Pathirana and Kaparakis-Liaskos, 2016). OMVs from *P. aeruginosa* stimulate the production of IL-8 in A549 human lung epithelial carcinoma cells (Bauman and Kuehn, 2012).

As already mentioned, interaction between the host vesicles and the microbiome in the intestine have been described. It was observed that the MVs contribute with gut homeostasis by enhancing innate immunity, since OMVs of the microbiota are involved in the activation of NOD1 signaling pathways in intestinal epithelial cells (Cañas et al., 2018). In fact, it was demonstrated that OMVs from *B. fragilis* induce immunomodulatory effects and prevent colitis. Inside OMVs, capsular polysaccharide A was detected, which interacts with dendritic cells *via* TLR2, enhancing regulatory T cells and the production of cytokine IL-10 (Shen et al., 2012). However, it is also suggested that vesicles play a role in nutrient acquisition for the entire microbiota community, since they are enriched with hydrolytic enzymes, such as glycosidases and proteases (Elhenawy et al., 2014). The mechanism of vesicle content packaging has not yet been elucidated; however, it seems to be a regulated process. When the RNA profile of *Salmonella* OMVs was analyzed under different environmental conditions, it was observed that some mRNAs were enriched in OMVs when compared to intracellular fractions, reinforcing the concept that MVs packaging is not a passive process, but tightly regulated (Malabirade et al., 2018).

**Figure 1:**
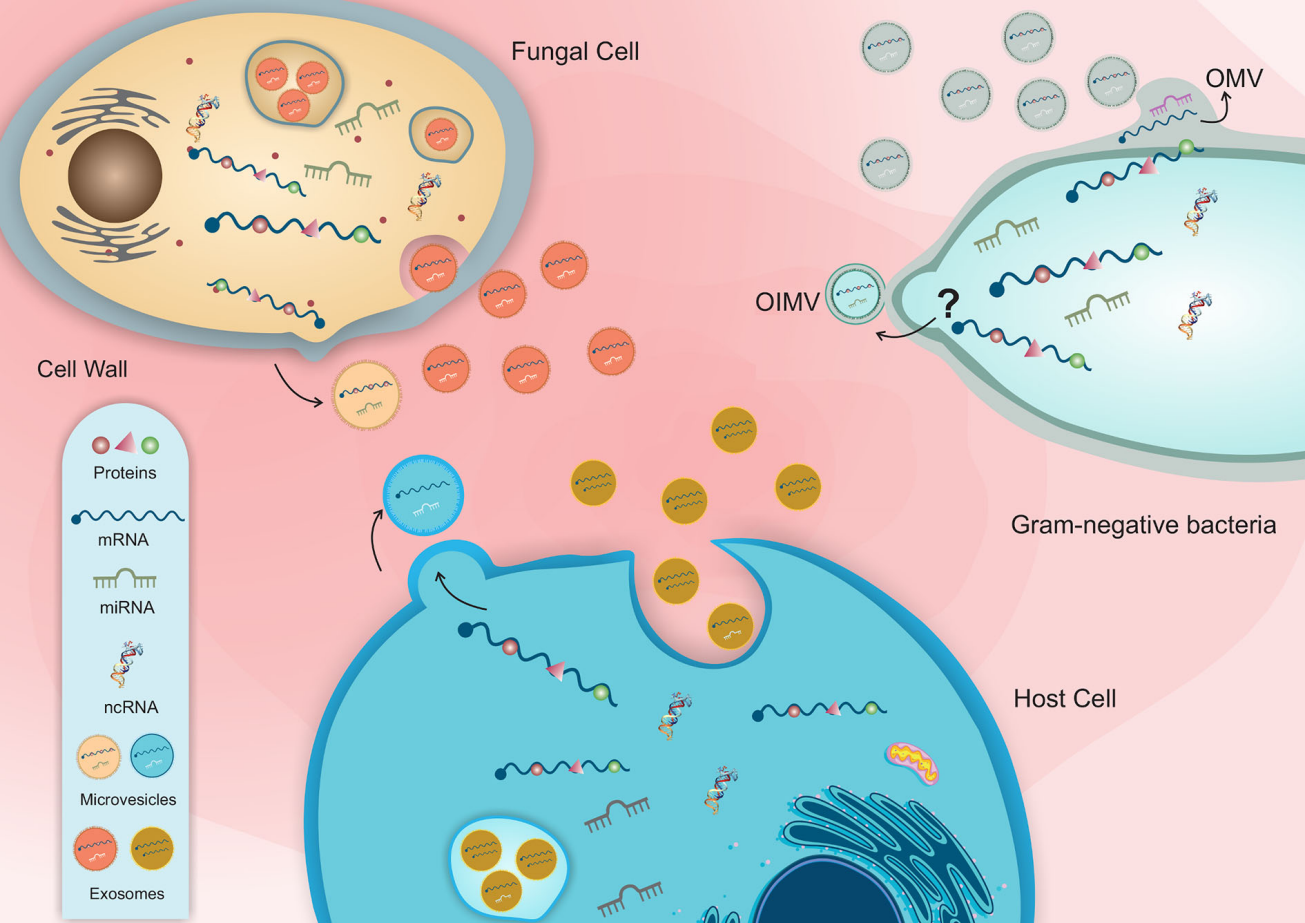
Schematic representation of host-pathogen EVs interaction and the distinct classes of RNA molecules in bacterial and fungal vesicles.

Bacterial MVs can transport different types of RNA molecules, including mRNA, tRNA, rRNA and ncRNA (Dauros-Singorenko et al., 2018) (**Figure 1** and **Table 1**). Studies characterizing the MV and extracellular RNA-content of *Escherichia coli* K-12 substrain MG1655 (OMVs and OMVs-free) identified mainly tRNA and rRNA fragments (3S rRNA, 16S rRNA, and 5S rRNA), as well ncRNAs (Ghosal et al., 2015). In OMVs from *Vibrio cholerae* strain A1552, ncRNA was the most abundant class of RNA identified (Sjöström et al., 2015). In *V. cholerae* O395, the enrichment of rRNA and sRNA, including CsrB1, CrsB2 and CrsB3 was also observed (Langlete et al., 2019).

**Table 1:** Summary of the role of RNA molecules present in extracellular vesicles on pathogen and host communication.

Origin	RNA molecule	Function	Reference
Host to pathogen	miR-199b-5p, miR-125b-5p	Those miRNAs were able to differentiate healthy individuals from septic and predict their prognosis	Reithmair et al., 2017
	miR-1224, miR-2146, miR-2134, miR-483, miR-710, miR-2141, miR-720, miR-155 and miR-34c	Human and mice miRNAs were able to enter bacterial cells and regulate gene expression	Liu et al., 2016
	sRNAs	*Arabidopsis* secretes EVs to deliver sRNAs into fungal cells to silence virulence-related genes	Cai et al., 2018
Pathogen	ncRNAs, tRNAs and rRNAs (3S rRNA, 16S rRNA, and 5S rRNA)	*Escherichia coli* K-12 OMVs can carry distinct types of RNA molecules	Ghosal et al., 2015
	ncRNAs, rRNAs and sRNAs (CsrB1, CrsB2 and CrsB3)	*Vibrio colerae* strain A1552 carries a great variety of ncRNA molecules	Sjöström et al., 2015; Langlete et al., 2019
	mRNAs	*Salmonella enterica* Serovar Typhimurium mRNAs were diferrentially enriched in OMVs	Malabirade et al., 2018
	microRNA-like molecules	Those molecules were identifeid in OMV from *E. coli* and *Streptococcus*	Lee and Hong, 2012; Kang et al., 2013
	microRNA-like, snoRNA snRNAs and mitochondrial tRNAs	Those RNA molecules were identified in EVs from *P. brasiliensis, C. neoformans, C. albicans* and *S. cerevisiae*	Peres da Silva et al., 2015
	mRNAs	mRNAs habe been found in fungal EVs which were involved in essential processes for survival and pathogenesis	Peres da Silva et al., 2015
	anti-sense ncRNAs and tRNAs	In *Histoplasma capsulatum*, anti-sense ncRNAs were found inside EVs	Alves et al., 2019
Pathogen to host	rRNAs, sRNAs	The RNA content of Escherichia coli strain 536 OMVs was found inside the recipient cell, reinforcing the hypothesis of inter-kingdom communication	Blenkiron et al., 2016
	sRNA (sRNA52320)	This sRNA led to a reduction in IL-8 secretion in the host cell	Koeppen et al., 2016
	sncRNAs sR-2509025 and sR-989262	In *Helicobacter pylori*, these ncRNAs reduced the LPS-mediated induction of IL-8 protein secretion in the host cell.	Zhang et al., 2020
	sRNAs	In periodontitis-causing bacteria, sRNAs in OMVs led to decreased levels of IL-5, IL-13 and IL-15	Choi et al., 2017
	sRNAs and allergens	*Malassezia sympodialis*’ EVs carry allergen molecules and sRNAs to host cells	Rayner et al., 2017
	RNA content	EVs from a more virulent strain of C. gatti increased the proliferation of a less virulent strain inside the macrophages	Bielska et al., 2018
	mRNAs	*Paracoccidioides* mRNAs present in the EVs could be actively translated	Peres da Silva et al., 2019

Blenkiron and coworkers demonstrated that bacterial MVs RNA content is transferred to the recipient cell and directed to the cytoplasm of epithelial cells and could be also found in the nucleus, reinforcing the hypothesis of inter-kingdom communication (Blenkiron et al., 2016). In fact, MVs also show regulatory activity in the host through MV-RNA molecules that are functional in the host’s cell. RNA sequencing analysis identified “microRNA-like” in *E.* coli and *Streptococcus mutans,* speculating that these small RNA molecules may play a role in bacteria similar to miRNAs in eukaryotes (Lee and Hong, 2012; Kang et al., 2013). As mentioned, MVs can trigger immune response in the host, however, in *P. aeruginosa* it was identified an interaction between an sRNA molecule present in the OMV and the host cell that led to a reduction in IL-8 secretion (Koeppen et al., 2016). Using RNA-Seq, Koeppen and coworkers identified the sRNA5320 present in OMVs from *P.* *aeruginosa*. Different assays were performed *in vitro* and *in vivo*, showing that this sRNA is transferred to cells *via* OMVs and plays a role in modulating the immune response by decreasing IL-8 secretion. Similarly, in *Helicobacter pylori*, sncRNAs sR-2509025, and sR-989262 can reduce the LPS-mediated induction of IL-8 protein secretion in human gastric adenocarcinoma cells (Zhang et al., 2020).

In addition, distinct sRNAs have been identified in the vesicles of three different bacteria that cause periodontitis. The transfection of synthetic copies of these sRNAs into Jurkat T cells resulted in decreased expression levels of IL-5, IL-13, and IL-15 (Choi et al., 2017). Afterwards, for *Aggregatibacter actinomycetemcomitans*, which is one of the causes of periodontitis, it was observed that the sRNA of OMVs can be incorporated by the host's RISC system, contributing to the alteration in gene expression of the host (Han et al., 2019). The potential of sRNAs to act in the pathogen-host interaction has been proposed as a target to be explored for the discovery of new biomarkers for bacterial diseases (Wang and Fu, 2019).

### Fungi EVs and the RNA Content

EVs have been described in several fungal species, both in yeast and in filamentous fungi such *Saccharomyces cerevisiae* (Zhao et al., 2019), *Candida albicans* (Vargas et al., 2015), *Histoplasma capsulatum* (Albuquerque et al., 2008), *Paracoccidioides brasiliensis* (Ganiko et al., 2011), *Cryptococcus neoformans* (Rodrigues et al., 2007), *Aspergillus fumigatus* (Souza et al., 2019), *Sporothrix brasiliensis* (Ikeda et al., 2018), *Malassezia sympodialis* (Rayner et al., 2017) and *Alternaria infectoria* (Silva et al., 2014).

As described for mammalian cells, fungal EVs are also classified according to their biogenesis, being able to release both exosomes and microvesicles (Kwon et al., 2019). The EVs, biogenesis process in fungi and how the sorting of their cargo occurs remains unknown. However, there are preliminary studies that report the relevance of EVs regulation pathways in molecular traffic across the cell wall (Bielska et al., 2018). In *C. albicans* EVs are key players to biofilm matrix production. ESCRT (endosomal sorting complexes required for transport) defective mutations caused reduced EV production and consequently biofilm thickness reduction and increased sensitivity to the antifungal drug (Mitchell et al., 2018). Mutations in genes that encode components of ESCRT machinery in *S. cerevisiae* also led to the reduction in EVs population and changes in the EVs proteomic profile (Zhao et al., 2019). The deletion of Sec6, a component of the exocyst complex involved with vesicles fusion with the plasma membrane, prevents EVs production and laccase secretion to extracellular milieu, and decreased virulence in mice (Panepinto et al., 2009). In addition, mutations in protein of the ESCRT complex, Vps27, led to MVB accumulation and decreased laccase transport to the cell wall (Park et al., 2020).

Fungal EVs have been related to several functions, such as biofilm matrix production (Mitchell et al., 2018), the delivery of virulence factors (Bielska et al., 2018; Ikeda et al., 2018; Konečná et al., 2019), cell wall remodeling (Zhao et al., 2019), host response and host–pathogen interaction (Rayner et al., 2017; Bitencourt et al., 2018; Johansson et al., 2018) (**Figure 1** and **Table 1**). The EVs are composed of polysaccharides (Rodrigues et al., 2007), lipids (Vallejo et al., 2012), allergens (Johansson et al., 2018), pigments (Frases et al., 2009), cytosolic and membrane proteins (Gil-Bona et al., 2015), and nucleic acids such RNA molecules (Peres da Silva et al., 2015; Rayner et al., 2017; Alves et al., 2019; Peres da Silva et al., 2019).

Several studies have identified different RNA species loaded in fungal EVs that could perform different functions in recipient cells. The commensal yeast *Malassezia sympodialis*, which colonizes human skin and is associated with common skin disorders, can secrete vesicles ranging from 50 to 600 nm, which carry allergens related to inflammatory responses and small RNAs ranging from 16 to 22 nucleotides in length (Rayner et al., 2017). For the human pathogenic fungi *P. brasiliensis*, *C. neoformans*, *C. albicans*, and *S. cerevisiae* EVs, several miRNA-like sequences, as well as small nucleolar RNAs (snoRNAs) and nuclear RNA and mitochondrial tRNAs, were identified in high abundance (Peres da Silva et al., 2015). Messenger RNAs have also been found in fungal EVs; they are involved in essential processes, such as vesicle-mediated transport, metabolic pathways, cellular responses to stress, transcriptional regulation and cell cycle control (Peres da Silva et al., 2015). The pathogenic fungi *H. capsulatum* also produces EVs enriched with different ncRNA populations; the most abundant were tRNA fragments. It was also identified anti-sense ncRNAs, with 25 nt in length, that aligned with specific regions of the transcripts and could act in gene expression regulation as a silencing mechanism (Alves et al., 2019). In fungi, ncRNAs are important players in gene expression regulation in the fungal cells. For example, during the transition phase in dimorphic fungi, a process already recognized as important in the context of infection and virulence. Antisense transcription of ncRNAs have been linked to hyphae and spore formation in *Ustilago maydis*, and the loss of such RNAs resulted in virulence attenuation (Morrison et al., 2012).

In an elegant work performed by Bielska and coworkers, it was shown that EVs from a more virulent strain of *C. gatti* could increase the proliferation of less virulent fungal cells inside the macrophages, thus promoting pathogen survival instead of clearance by the host cell. The protein and RNA fractions were required for this transference process (Bielska et al., 2018).

Although it is not clear whether EV mRNAs are translated into functional peptides, the presence of functional mRNAs in fungal EVs was confirmed from two species of *Paracoccidioides* by *in vitro* translation (Peres da Silva et al., 2019). The presence of these transcripts suggests that they can be internalized by fungal or host cells and alter gene expression regulation and play a role in the host-fungal interaction. Most of the mRNAs present in the EVs are associated with virulence, like heat shock proteins Hsp 70 and Hsp 90-like, that have a role during infection of dimorphic fungi (Peres da Silva et al., 2019). It is possible to speculate that EV mRNA can be translated into the host cell, inducing gene expression alterations that could aid pathogen infection and survival.

Small RNAs can induce gene silencing by binding to argonaute proteins, directing the RNA-induced silencing complex (RISC) to target mRNAs for their repression. In host and pathogenic fungi interactions, sRNA molecules can participate in cross-kingdom communication (Weiberg et al., 2013; Chen et al., 2014; Weiberg et al., 2015; Cai et al., 2018). In plants, the EVs can act as effectors that suppress fungal pathogens (Cai et al., 2018). Phytophogenic (Weiberg et al., 2013; Chen et al., 2014; Wang et al., 2016) and entomopathogenic fungi (Cui et al., 2019) interfere in plant and insect immunity, respectively. They play a role in silencing host immunity genes by hijacking the host's RNAi machinery to facilitate infection (Weiberg et al., 2013; Wang et al., 2016). The entomopathogenic fungus *Beauveria bassiana* exports miRNA-like molecules loaded in vesicles to the host's mosquito *Anopheles stephensi*, attenuating host immunity, and facilitating infection (Cui et al., 2019).

Conversely, the host can also suppress fungal pathogenesis. The extracellular vesicles of plants play an essential role in sRNA trafficking between *Arabidopsis* and the pathogen *Botrytis cinerea*. *Arabidopsis* secretes EVs to deliver sRNAs into fungal cells to silence virulence-related genes (Cai et al., 2018).

## Concluding Remarks

In summary, this unconventional pathway of communication is gaining more attention because it is involved in all aspects of cell life regarding its important, if not essential, role in organism homeostasis. Regarding infection, it was shown that EVs of both hosts and pathogens play a role in either promoting or fighting it where the EVs influence antigen presentation, immunological stimulation and suppression as well as transmission of virulence factors. Additionally, one of the most widely studied aspects of EVs is their RNA cargos, mainly because of their capacity to regulate gene expression in the recipient cell, the microRNA pathway, leading to gene silencing that can favor the pathogen, and also the mRNA in the EVs that can be translated and influence the cell metabolism. In addition, some physiological aspects regarding pathogenesis and host response were studied and are helping to understand how the EVs can be used for diagnostic and therapeutical purposes, the RNA present in EVs derived from bacteria and fungi and their role in the host are recent and still need to be further addressed for most of the work has been descriptive. The EV biogenesis and how specific molecules are sorted and directed to them are questions to be answered. Nevertheless, the promising results obtained so far are paving a new path for the study of RNA present in the vesicles and their important role in cell communication and gene expression regulation highlighting the potential of EVs and their role in the host during infection.

## Author Contributions

IM, RA, AL, HF, and LA discussed, wrote, and approved the manuscript in its current form. All authors contributed to the article and approved the submitted version.

## Funding

This work received financial support from Inova Fiocruz/Fundação Oswaldo Cruz [Grant number VPPCB-07-FIO-18-2-52] and CNPq [Grant number 442317/2019-0]. LRA is a research fellow awardee from CNPq.

## Conflict of Interest

The authors declare that the research was conducted in the absence of any commercial or financial relationships that could be construed as a potential conflict of interest.
